# PORTALS: design of an innovative approach to anticoagulation management through eHealth

**DOI:** 10.1186/s12913-017-2142-2

**Published:** 2017-03-16

**Authors:** E. P. W. A. Talboom-Kamp, N. A. Verdijk, I. J. S. H. Talboom, L. M. Harmans, M. E. Numans, N. H. Chavannes

**Affiliations:** 1 Public Health and Primary Care Department, Leiden Universitair Medisch Centrum (LUMC), P.O. Box 9600, Leiden, 2300 RC The Netherlands; 2 Saltro Diagnostic Centre, P.O. Box 9300, Utrecht, 3506 GH The Netherlands; 3 Foundation Zorgdraad, Wijnand van Arnhemweg 54, Oosterbeek, 6862XN The Netherlands

**Keywords:** eHealth, Self-management, Self-monitoring, Venous thromboembolism, Atrial fibrillation, Anticoagulant therapy, e-learning

## Abstract

**Background:**

For the monitoring of International Normalized Ratio (INR) values, venous thromboembolism (VTE) and atrial fibrillation (AF) patients can visit anticoagulation clinics, laboratories, or physicians for venous puncture. Point-of-care testing (POCT) made it possible for patients to monitor INR themselves (self-monitoring) and even self-adjust their medication dosage (self-dosage). Both skills are accepted as forms of self-management. eHealth applications can improve this self-management, resulting in better clinical outcomes.

**Methods:**

Our study, called PORTALS, aims at identifying the optimal implementation strategy of training to improve self-management and explore factors that enhance good self-management skills. In addition, the relationship between the implementation strategy of training, clinical outcomes, and individual characteristics will be investigated. Of the 247 recruited participants, 110 chose to continue with regular care. 137 patients have been randomly divided in subgroups and compared using a parallel cohort design: one group will be trained and educated by e-learning, and the other group will receive face-to-face group training.

**Discussion:**

More insight in factors that enhance good self-management will help to improve clinical outcomes and patient satisfaction on anticoagulation therapy. Our study will provide practical insights and knowledge of eHealth in daily practice and of the importance of education on the adoption of self-management. We expect the self-management program including training to help patients to better manage their own INR values and medication use, thereby increasing health status and diminishing thromboembolic events and hospitalisation.

**Trial registration:**

The Netherlands National Trial Register, number NTR3947.

## Background

Venous thromboembolism (VTE) and atrial fibrillation (AF) are common causes of mortality and morbidity, and are associated with high medical costs [1]. With the ageing population and persisting unhealthy lifestyles, the prevalence of VTE and AF is rising rapidly [2]. Treatment of VTE and AF patients consists of, among other interventions, anticoagulant therapy (AT) with vitamin K-antagonists to treat, slow down, or prevent formation of blood clots [2]. AT demands frequent monitoring of blood samples to measure its effects. Results are expressed as International Normalized Ratio or INR values. In 2009 there were over 385.000 patients in the Netherlands who were treated with anticoagulants, more than half of whom suffered from AF (211.000 patients) [3]. Between 2005 and 2009 the number of VTE patients in the Netherlands increased by 13%; the prevalence of AF patients increases every year, especially amongst males due to the rising survival rate of myocardial infarcts [4].

For the monitoring of INR, patients have to visit anticoagulation clinics, laboratories, or physicians for venous puncture. This is one of the major disadvantages of anticoagulant therapy with vitamin K antagonists and, together with frequently occurring unstable anticoagulation, one of the reasons that other anticoagulants (novel oral anticoagulants, NOACs) are being developed and often preferred as an alternative [5]. However, since the alternatives show disadvantages and risks too, improving procedures around vitamin K antagonists attract attention [6].

Point-of-care testing (POCT) is one of these potential improvements: it generates rapid test results and has been widely implemented in the last decade. POCT made it possible for patients to monitor INR values themselves (self-monitoring) and, as a next step, to even self-adjust their medication dosage (self-dosage). The major benefit of this self-management approach is the increased involvement of patients in their own care process, thus resulting in improved adherence to AT, and thereby reduction of complications. Research showed that self-management in AT patients decreases the risk of thromboembolic complications and mortality at a constant frequency of bleeding complications [7]. Several published studies and systematic reviews have suggested methods of monitoring anticoagulation therapy may be equal to or better than standard monitoring by a physician. Patients who self-monitor or self-dose can improve the quality of their oral anticoagulation therapy [8]. When patients start with self-monitoring or self-dosage, they receive structured education and training in self-testing and adjustment of medication dosage. In addition, eHealth applications are developed in the form of online web portals to share self-measured INR values with care professionals. These web portals also provide programs that support self-adjustment of medication dosage. Research showed that self-management with online support results in improved INR values (Time in Therapeutic Range, TTR, of 10–23%) compared to self-management without online support (less than 4% improvement of TTR) [9, 10]. Moreover, patient satisfaction is higher when using online remote monitoring of INR [11]. Based on these results we conclude that eHealth applications do improve self-management of vitamin K antagonist anticoagulant therapy, resulting in better clinical outcomes.

In the last few decades, self-management has become an important strategy for coping with chronic illnesses. Adequate self-management requires the individual ability to deal with symptoms, treatment, and physical and social consequences of a disease. The basic principle of self-management is that behavioral change cannot succeed without patients taking their responsibility [12]. Research on other chronic diseases such as diabetes [13], COPD [14], and heart failure [15] showed that personality aspects such as self-efficacy are important factors in successful self-management. This notion is derived from the social cognitive theory, which states that behavioral change is made possible by a personal sense of control. Self-efficacy has been described as the “belief in one’s capabilities to organize and execute the course of action required to produce given attainments”.

As education is the basic approach in development of self-management skills, we expect that the strategy to implement the educational support largely affects the individual level of self-management and thereby clinical outcomes. However, it is clear that self-management is not a ‘one-size-fits-all’ intervention. How can self-management skills then be optimized in a large groups of individuals, such as patients with AT? Which factors positively influence the acceptance of self-management? To answer these questions for patients with AT we designed the PORTALS study.

Our goal is to analyze the effect of the implementation of e-learning versus a group training on top of a program of self-monitoring or self-management. In addition we will investigate the relationship between the implementation strategy of training, clinical outcomes, and individual patient characteristics.

We consider both self-monitoring and self-adjustment of medication as important self-management skills. In this study we defined self-management skills as the usage of the self-management platform; the amount of login sessions. For this definition, the difference between self-monitoring and self-dosage is not relevant. Self-monitoring and self-dosage activities are registered within the same login session. Therefore, we make no distinction between patient-self-dosage and patient-self-testing in this study. All skills are accepted as forms of self-management.

## Methods

In this study, self-management will be offered to patients of the Saltro Thrombosis Service (outpatient anticoagulation clinic and laboratory), who currently receive usual care for long-term AT.

Usual care consists of frequent monitoring of blood samples to measure the effects of vitamin K-antagonists. These blood samples are taken by classical venipuncture at primary care locations or at home. Based on the INR, specialized medical doctors define the acquired dosage for individual patients and follow puncture; patients receive the advice and instruction on individual basis.

**Fig. 1:**
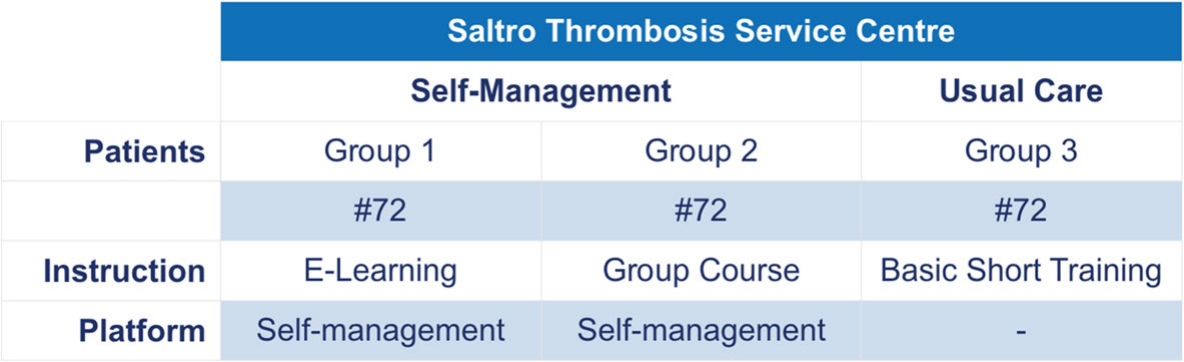
Subdivision within the Saltro Thrombosis Service into three groups; Groups 1 and 2 perform self-management; group 1 was instructed by e-learning and group 2 by group course. Group 3 receives the usual care, with brief instructions

We used a parallel cohort design to investigate determinants of optimal implementation; we will compare the different training methods. After inclusion, participants will be randomly divided in subgroups: one group will be trained and educated by e-learning (group 1) and the other group will receive face to face group training (group 2). Both the e-learning and the group training consist of at least three components: i) disease-specific knowledge of VTE; ii) self-testing skills; iii) use of the web portal; and iv) self-adjustment of medication. The fourth module is voluntary. Hence, 2 groups of self-management patients will be formed. Patients who do not wish to start with self-management will be invited to participate in the non-self-management group, a parallel cohort group describing usual care (group 3). They continue to receive regular care of high quality. In Fig. 1 the inclusion of the research groups is summarized.

Due to the nature of this study (implementation research in a real-life care setting) a perfect RCT design is not feasible: patients cannot be forced to start self-management. Patients will however be randomized in group 1 (e-learning) and group 2 (group training). Furthermore, patients unable to dose their medication are free to continue with only self-monitoring. This study will be performed using a parallel cohort design. Although bias may occur, the results of this study remain valuable because in daily practice biases will occur too when it comes to patients voluntary choosing the type of care they are willing to receive. Based on our study design, comparison between e-learning and group training for self-management (group 1 and 2) and non-self-management patients (group 3) is applicable considering specific conditions in the choice of the statistics. Parallel cohort group 3 provides valuable information about patients who are unable or unwilling to use online supported self-management programs.

### Power calculation

In thrombosis patients clotting speed is expressed in INR values. Optimal INR values vary between individual norms. Therapeutic effects are expressed as the percentage of Time the patient is in the Therapeutic INR Range (TTR). A pilot study of 100 patients of the Saltro Thrombosis Service Centre shows that the average TTR is 84% (SD 10). Research shows that TTR improves significantly in self-management compared with usual care, with 5–13% increasing TTR [16]. To prove a relevant effect of the new implementation strategy of e-learning or group training (>5%) at a power 80% and α = 0.05, 63 patients must be included per group. Taking into account a 15% drop out, 72 (63/0.85) patients are needed per study group.

Previous studies reported a 21–77% response at inclusion of study [8, 9, 17]. Even if attrition rate at inclusion would be only 21%, there are sufficient eligible patients to reach an adequate sample size: at 21% attrition rate, 343 patients (72/0.21) must be approached per group to include sufficient participants in the study. As this study includes three research groups, a total of at least 1029 patients is needed to complete the study. Currently, 8950 patients receive usual care from the Saltro Thrombosis Service.

### Participants

This study focuses on patients of the Saltro Thrombosis Service who voluntary choose to start with self-management. The Thrombosis Service states several inclusion criteria for their patients to start with self-management. The first criterion is a long-term indication for anticoagulants as the training period and investments exceed the regular three to six months of short-term anticoagulant prescription. In 2013, 85% of the patient population of the Thrombosis Service suffered from long-term indication for anticoagulants. Secondly, internet access is mandatory as monitoring of patients by the Saltro Thrombosis Service is provided by the web portal. In the Netherlands 95% of the people have internet access at home. The average age of patients of the Saltro Thrombosis Service is 65 years. In 2013, 61% of Dutch people over 65 used internet daily and over 75% used it during the last three months [18]. Of people aged between 55 and 65, 81% used internet on a daily basis [19]. Third, one must have stable INR values: during a period of at least five days at least two INR values must be within therapeutic range. Patients who meet the criteria for self-management will be approached for participation in the study. Because self-management is already a regular care process of the Saltro Thrombosis Service, the group training is also available for people who are not willing to enter the study. The e-learning is dedicated for participants of the study as this is a new implementation method.

### Recruitment of patients

Patients of the Saltro Thrombosis Service who receive regular care will be recruited. In 2013, 8950 patients received usual care from the Saltro Thrombosis Service, of which 85% with long-term indications. A random selection of 1632 patients was approached for participation in the study using three methods:

1. First a randomly selected group of 475 patients was informed by letter. Of these, 233 patient responded and 59 were willing to participate in the study.
2. Second we approached patients through personal invitation by nurses of the Saltro Thrombosis Service who collect blood by venipuncture. All nurses were trained by the research team to inform patients well about the study and research goals. During five months, nurses actively informed 692 patients about self-management. Of those, 139 patients were interested to participate in the study. In addition, 234 were interested in self-management too, but not in participation in the study.
3. Third we recruited patients by telephone. Two trained research assistants called patients at home to inform them about the possibilities of self-management and about the study. By telephone 465 patients were approached, of which 111 were willing to participate in the study. In addition, 52 patients signed up for self-management but were not willing to participate in the study.

**Fig. 2:**
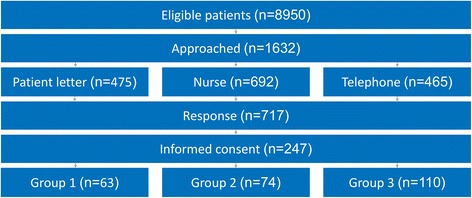
Schematic summary of recruitment; A schematic summary of the number of eligible patients, how many of them were approached, with what method they were approached, how many responded, how many are participating, and in which group

Participants were only included in the study after written informed consent was received. Because not all patients signed an informed consent, 247 participants were finally included. Of these, 110 continued to receive regular care and 137 patients were randomly divided in group 1 and 2 using a computer program. In group 1 (e-learning) 63 patients were included and in group 2 (group training) 74 patients were included. In Fig. 2 recruitment is summarized.

Patients who are willing to participate will receive rewards: a € 25,- gift card after completing the first set of questionnaires and a € 50,- gift card after completing the last set of questionnaires after 18 months.

### Responder analysis

The total population of the Saltro Thrombosis Service on January 1^st^, 2014 was 10.209 patients of which 8.939 patients received regular care (eligible patients) and 1.271 self-management (894 self-monitoring and 377 self-dosage). 53% were men and 47% women. The mean age was 73 years (SD 14). In the study population 247 patients were included of which 72.9% were men and 27.1% women. The median age was 66.9 (IQR 59.5–72.7) years in the total group; 65 years in group 1 and 65.8 years in group 2, and 69.6 years in group 3.

**Fig. 3:**
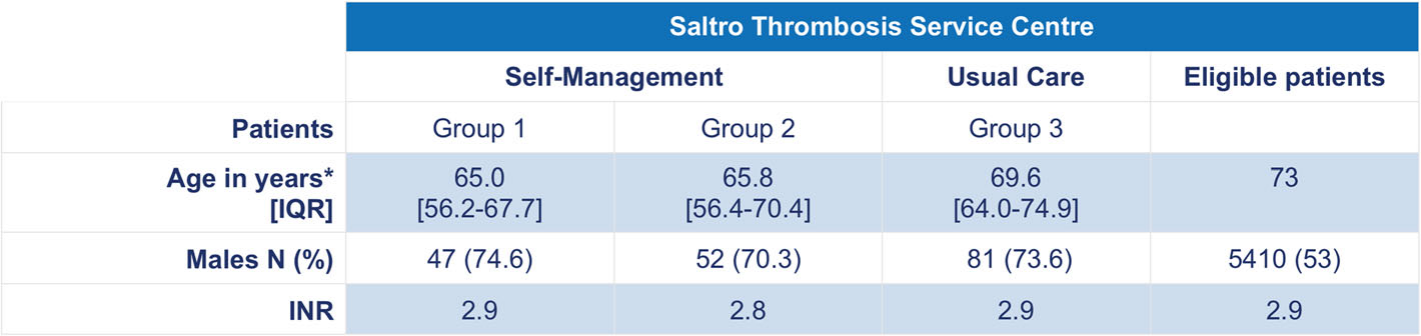
Characteristics of eligible patients versus included patients; The average age, percentage of males, and average INR of eligible patients versus included patients is shown

Analysis showed a significant difference in median age between the groups. Younger men participated in the PORTALS trial compared to patients who declined participation. The average INR of the participants did not significantly differ from the eligible population at the start of the study. In Fig. 3 the characteristics of the patients are summarized.

Among patients who were recruited by letter we asked non-participants to inform us about their reasons. 35 patients filled in the non-responders form 12 patients did not want to start self-management because they had no computer or internet access, 7 patients were satisfied with regular care and wanted to continue it, 6 patients felt they were too old, 10 patients had other reasons such as painful fingers (1), physical inabilities (2), training is too much effort (2), insecurity (2) or unknown reasons (3).

### Allocation procedure

Participants were randomly selected for e-learning (group 1) or group course (group 2) during the registration procedure for the training by a computerized program. Participation in group 3 was not randomized but free choice for patients.

In Fig. 2 the total amount of included patients is summarized.

### Intervention

This study investigates the effect of different implementation strategies on adequate training for self-management skills in patients with AT expressed in TTR, as well as potential determinants of the effect. We compare group training versus e-learning. Patients who follow e-learning have to pass at least three modules. The first module contains general education about anticoagulation including test questions. Patients can only successfully pass this first module after all questions are answered correctly. They then receive a written confirmation. With this confirmation patients can pick up a self-testing device at one of the Saltro Thrombosis Service locations. Furthermore, patients get access to the second module that teaches them to use the self-testing device. After one or two weeks patients have an appointment with a nurse of the Thrombosis Service Centre to show their self-testing skills. If the skills are sufficient, the nurse explains the patients how to use the online web portal. The following three months a training period is applicable, during which patients measure their INR weekly and inform the Thrombosis Service using the web portal. After three months, patients have another control appointment to check their self-testing skills technique and adequate use of the web portal. If patients are able to provide reliable INR values by self-measurement and if they adequately use the web portal to inform the Thrombosis Service about testing results and relevant medical information, the self-monitoring training is ended. From that moment on, patients have control appointments with the nurse every six months. In addition, patients are offered to continue with training in self-adjustment of medication dosage. This training is only available in a group course.

The group training consists of two meetings. During the first meeting patients are trained to use the device to measure their own INR and they are trained to use the web portal. Patients practice at home for three weeks. At the second meeting the capillary technique of patients is checked by nurses of the Thrombosis Service. In addition, patients receive general education about anticoagulation. The following three months a training period is applicable during which patients measure their INR weekly and inform the Thrombosis Service using the web portal. After three months patients have a control appointment with the nurse to check their capillary technique and to discuss the training period. If patients are able to provide reliable INR values by self-measurement and if they adequately use the web portal to inform the Thrombosis Service about testing results and relevant medical information, the self-management training is ended. From that moment on, patients have control appointments with the nurse every six months. In addition, patients are offered to continue with training in self-adjustment of medication dosage. This training is only available in a group course.

The regular care group receives traditional thrombosis care, which means a basic instruction by nurses of the Saltro Thrombosis Service in their own home. Patients are explained what locations they can go to for venous puncture. Based on analyses of the blood samples (INR values) professional thrombosis doctors give written instructions about medication dosage and lifestyle to the VTE patients. This also includes indication for follow-up puncture.

**Fig. 4:**
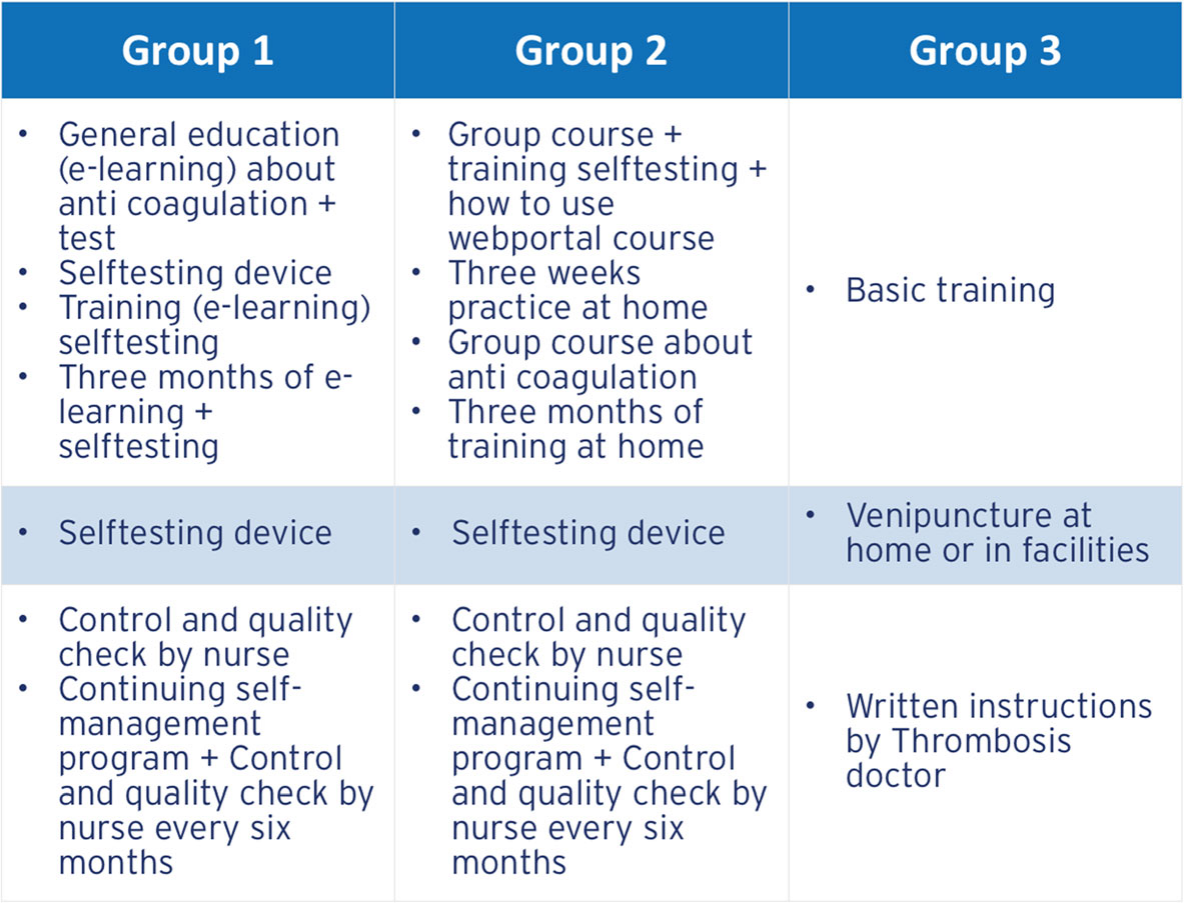
Time course for group 1, 2 and 3; The three groups and the time courses of their trainings, blood testing procedures, and instructions

All training schedules are summarized in Fig. 4.

### Data collection

**Fig. 5:**
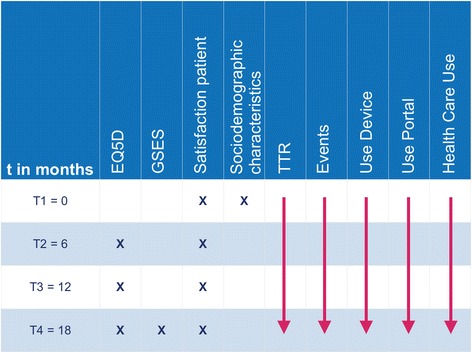
Measurements over time; A schematic overview of the different types of measurements on T1 = 0, T2 = 6, T3 = 12, and T4 = 18 months

The data collection consists of questionnaires to measure the determinants and secondary outcomes, that patients of group 1 and 2 receive by e-mail. Patients in research group 3 (who do not work with the web portal) receive the same questionnaires either by e-mail or post. In case of non-response we will send two reminders by mail or email and will approach the patients once by telephone. In Fig. 5 planning of measurements is summarized. There are four measurements in this study during a period of 18 months. INR values and thromboembolic events are monitored and registered daily by professionals of the Saltro Thrombosis Service. In addition health care use, the number of self-tests and use of the portal are continuously registered in the web portal. Data collection is summarized in Fig. 5.

### Outcome parameters

At first we compare clinical outcomes (TTR) and complications between three groups; second we compare TTR, complications and self-management skills of two different implementation methods in group 1 and 2. Second, the relationship between self-management skills, clinical outcomes, and individual characteristics will be investigated.

#### Primary outcome

Health status is the primary outcome, expressed by TTR and severe complications (bleedings and thromboembolic events). TTR is computed as INR values within therapeutic range based on individual targets, calculated with the Rosendaal method [20]. INR values will be measured using a whole blood PT/INR monitor (CoaguChek, Boehringer Mannheim, Germany). Bleedings and thromboembolic events will be registered in patient files by professionals of the Saltro Thrombosis Service during the total study period of 18 months. Events can be reported by general practitioners and hospital professionals by e-mail or telephone, or by patients themselves using the online portal, e-mail, or telephone.

In this study, adequate self-management skills are defined as a) the ability to correctly measure one’s own INR; b) the ability to use the online web portal to report all measured INR values; c) the ability to use the online web portal to report all relevant medical conditions and d) the ability to correctly calculate dosage schedules. The latter being only applicable if patients voluntary choose to self-adjust their own medication intake. Correct self-testing of INR will be judged by thrombosis nurses during yearly control visits. We registered the usage of the self-management platform, including sharing of information and calculation of medication dosage; the amount of login sessions will be used as a measure for self-management skills. The usage counts will be analyzed.

#### Determinants

We expect that the relationship between the level of self-management skills and clinical outcomes is influenced by socio-demographic, (psycho) social characteristics, and self-efficacy.

*Self*-*efficacy* is measured using the Generalized Self-Efficacy Scale (GSES), which will be displayed at baseline. This 10-item questionnaire was designed in 1981 by Jerusalem en Schwarzer [21]. The items are scored by a four-point scale on which a higher score reflects higher self-efficacy. Research in 28 countries showed that Cronbach’s alpha varies between .76 and .90, of which mostly above .80 [22].

*Socio*-*demographic characteristics* will be assessed by a purpose-designed questionnaire (online). As use of the web portal is part of the self-management program and as decreased access to internet and low general health outcomes have been associated with lower socioeconomic status, minority racial/ethnic groups, older age, and poorer health we will also include the following characteristics in our questionnaires: age, socioeconomic status, marital status, and general use of online and digital products and services.

#### Secondary outcomes

*Quality of life* (QoL) is assessed using the EuroQol-5D (EQ-5D). This questionnaire contains 5 items with a 3-point Likert scale. A higher score reflects a higher quality of life. The EQ-5D comprises 5 levels: mobility, self-care, daily activity, pain/discomfort, and anxiety/depression. Research showed that the EQ-5D is a reliable and valid questionnaire [23]. The EQ-5D can be used to compute QALY’s, which are necessary to evaluate cost-effectiveness.

*Direct costs* of both intervention methods will be included in this study. Intervention costs include development costs of e-learning and costs of group courses. Development costs are provided retrospectively by the owner of the portal. Implementation costs are administered by the research group, and mainly consist of personnel costs and practice materials.

*Health care use of the Saltro Thrombosis Service is constantly registered* in this study the health care use of the Saltro Thrombosis Service is constantly registered.

### Data analyses

1. Baseline characteristics between the 3 groups will be explored using Chi-square tests and Kruskal-Wallis tests.
2. To investigate the effect of different implementation methods of training versus the parallel cohort group, TTR and complications between groups will be analyzed using multilevel linear regression modelling (mixed models). First, outcomes will be compared between the three groups. A second analysis will be used to compare the difference in effect between e-learning and group training (group 1 vs group 2) on TTR, complications and usage using mixed models. Analyses will be adjusted for age and gender.
3. To examine the impact of GSES and education on the effect of different implementation methods, those variables will be included in the mixed models.

### Ethical principles

Increasing costs and deficit of health care professionals stress the urge for efficient health care processes. Benefits of online supported self-management regarding clinical effects have been repeatedly demonstrated, but extensive integration in clinical practice stays behind. This study aims to explore implementation methods for optimal integration of online supported self-management in primary care. Optimal integration stimulates patients in self-management and improves efficiency and accuracy administration and communication. We expect patients to improve their health status while decreasing health care use. On the other hand, self-management will not be offered to patients who are unable to use internet. They will however not be in disadvantage by receiving usual care. All over, this study will be conducted according to the principles of the Declaration of Helsinki (version 59, 2008) and in accordance with the Medical Research Involving Human Subjects Act (WMO).

## Trial status

PORTALS is an ongoing trial.

## Discussion

Adequate self-management requires the individual ability to deal with symptoms, treatment, and physical and social consequences of a disease. The basic principle is that behavioral change cannot succeed without patients taking their responsibility [24]. Education is an important factor for the enhancement of self-management skills; research showed that patients who understand more about their disease, health, and lifestyle have better experiences and health outcomes and often use less health care resources. The effect is even bigger when these patients are empowered to and responsible for managing their health and disease [25].

In this study we aim to empower patients with AT by providing self-management including a web portal and education. We expect this self-management program to help patients to better manage their own INR values and medication use, thereby increasing health status and diminishing thromboembolic events and hospitalisation. Health status (INR and TTR) is the main outcome. We investigate the optimal implementation strategy of training of self-management and the relationship with personality and characteristics to provide practical insights in determinants of successful implementation of patient self-management portals in real-life thrombosis care settings.

By analyzing the non-participation reasons (35 patients), we can conclude that the possession of a computer, age and satisfaction with usual care are the most important reasons not to start with this study. These aspects can create a bias in the individual ability of the whole self-management group of patients how to deal with their disease.

This PORTALS study has several strengths. The self-management in combination with a web portal and different forms of education are integrated in real life care settings and will therefore provide practical insights and knowledge of eHealth in daily practice. We expect to learn what type of education can be a significant factor in the adoption of self-management. Furthermore, this study adds Dutch evidence to the existing body of knowledge which is important because local political and financial factors have a major impact on successful integration in daily practice [26]. This study also has several limitations: from a technical perspective the development of the web portal is a difficult task due to lack of broad experience in the field. The content of the web portal will affect our outcomes, but is beyond the scope and influence of our study. From a human perspective, effects through self-management imply behavioural changes. Behavioural changes require time, whereas the study period is limited to 18 months.

## Abbreviations

AF: Atrial fibrillation
COPD: Chronic obstructive pulmonary disease
EQ-5D: EuroQol-5D
GSES: Generalized Self-Efficacy Scale
INR: International Normalized Ratio
NOACs: Novel oral anticoagulants
POCT: Point-of-care testing
RCT: Randomised controlled trial
SD: Standard deviation
TTR: Time in therapeutic range
VTE: Venous thromboembolism
WMO: Wet medisch-wetenschappelijk onderzoek met mensen (Medical Research Involving Human Subjects Act)
